# Helsinki University Hospital Personnel and Covid -19 Pandemic– a two-year follow-up of insomnia and psychological distress symptoms

**DOI:** 10.1192/j.eurpsy.2023.324

**Published:** 2023-07-19

**Authors:** T. Laukkala, T. Rosenström, K. Tuisku, K. Junttila, T. Haapa, P. Jylhä

**Affiliations:** Helsinki University Hospital, Helsinki, Finland

## Abstract

**Introduction:**

Covid -19 pandemic challenged health care personnel, especially frontline employees. The increased workload was unevenly distributed.

**Objectives:**

The aim of the present study is to assess potentially traumatic pandemic work-related events (PTEs), psychological distress and insomnia symptoms especially among employees who were in the frontline June 2020, after 24 -month follow-up.

**Methods:**

Participants were recruited from the Helsinki University Central Hospital personnel on June 2020 and followed via electronic surveys for 24 months. Mental Health Index 5 (MHI-5) and Insomnia Severity Index (ISI) was used to assess psychological distress and insomnia symptoms. Potentially traumatic events related to pandemic work (PTEs) were asked. The study is described in detail elsewhere (R1, R2).

**Results:**

On May 2022, early frontline employees from June 2020 (N=1171) continued to report a greater frequency of PTEs compared to those not in early frontline (N=3623) (19.4% vs. 9.5%; OR = 2.29, 95% CI = 1.51–3.46). They did not report statistically significantly greater frequency of psychological distress (14.2% vs. 9.9%; OR = 1.5, CI = 0.96–2.35), nor sleep problems (8.9% vs. 5.8%; OR = 1.57, CI = 0.91–2.72). The difference was not quite significant for the continuously varying MHI-5 scores either (p = 0.058 in t-test and p = 0.064 for Kruskal-Wallis test), but the continuous ISI scores at the last follow up were still statistically significantly higher for the early frontline employees than for the non-frontline employees (6.82 vs. 5.51; p = 0.001 in t-test and p = 0.001 in Kruskal-Wallis test). Attrition from the study was higher among the early frontline personnel compared to others. The odds of no show at 24-month wave were 1.42-fold for those at the frontline (95% CI = 1.2–1.68). This effect was not fully explained by age, sex, profession or having experienced a potentially traumatic event at the baseline.

**Conclusions:**

Early frontline employees who participated the 24 -month follow-up, did not report significantly more psychological distress than other employees. Subthreshold changes in insomnia scale are in line with a recent meta-analysis R3.

R1. Haravuori H, Junttila K, Haapa T et al. Personnel Well-Being in the Helsinki University Hospital during the COVID-19 Pandemic-A Prospective Cohort Study. Int J Environ Res Public Health. 2020 Oct 28;17(21):7905.

R2. Laukkala T, Suvisaari J, Rosenström T et al. COVID-19 Pandemic and Helsinki University Hospital Personnel Psychological Well-Being: Six-Month Follow-Up Results. Int J Environ Res Public Health. 2021 Mar 4;18(5):2524.

R3. AlRasheed MM, Fekih-Romdhane F, Jahrami H et al. The prevalence and severity of insomnia symptoms during COVID-19: A global systematic review and individual participant data meta-analysis. Sleep Med. 2022 Aug 8;100:7-23.

**Disclosure of Interest:**

None Declared

